# Adenosine Kinase Inhibition Protects against Cranial Radiation-Induced Cognitive Dysfunction

**DOI:** 10.3389/fnmol.2016.00042

**Published:** 2016-06-03

**Authors:** Munjal M. Acharya, Janet E. Baulch, Theresa A. Lusardi, Barrett. D. Allen, Nicole N. Chmielewski, Al Anoud D. Baddour, Charles L. Limoli, Detlev Boison

**Affiliations:** ^1^Department of Radiation Oncology, University of CaliforniaIrvine, CA, USA; ^2^R. S. Dow Neurobiology Laboratories, Legacy Research InstitutePortland, OR, USA

**Keywords:** adenosine, adenosine kinase, astrogliosis, radiation, cancer therapy, cognition, neuroprotection

## Abstract

Clinical radiation therapy for the treatment of CNS cancers leads to unintended and debilitating impairments in cognition. Radiation-induced cognitive dysfunction is long lasting; however, the underlying molecular and cellular mechanisms are still not well established. Since ionizing radiation causes microglial and astroglial activation, we hypothesized that maladaptive changes in astrocyte function might be implicated in radiation-induced cognitive dysfunction. Among other gliotransmitters, astrocytes control the availability of adenosine, an endogenous neuroprotectant and modulator of cognition, via metabolic clearance through adenosine kinase (ADK). Adult rats exposed to cranial irradiation (10 Gy) showed significant declines in performance of hippocampal-dependent cognitive function tasks [novel place recognition, novel object recognition (NOR), and contextual fear conditioning (FC)] 1 month after exposure to ionizing radiation using a clinically relevant regimen. Irradiated rats spent less time exploring a novel place or object. Cranial irradiation also led to reduction in freezing behavior compared to controls in the FC task. Importantly, immunohistochemical analyses of irradiated brains showed significant elevation of ADK immunoreactivity in the hippocampus that was related to astrogliosis and increased expression of glial fibrillary acidic protein (GFAP). Conversely, rats treated with the ADK inhibitor 5-iodotubercidin (5-ITU, 3.1 mg/kg, i.p., for 6 days) prior to cranial irradiation showed significantly improved behavioral performance in all cognitive tasks 1 month post exposure. Treatment with 5-ITU attenuated radiation-induced astrogliosis and elevated ADK immunoreactivity in the hippocampus. These results confirm an astrocyte-mediated mechanism where preservation of extracellular adenosine can exert neuroprotection against radiation-induced pathology. These innovative findings link radiation-induced changes in cognition and CNS functionality to altered purine metabolism and astrogliosis, thereby linking the importance of adenosine homeostasis in the brain to radiation injury.

## Introduction

The adverse neurocognitive side effects of radiotherapy used to treat CNS cancers are unintended and largely unavoidable. It is now well-documented that major changes occur in the brain following exposure to clinical radiotherapy protocols, including severe morphologic and physiological damage that coincides with substantial depletion of CNS stem cell populations (Monje et al., 2002; Mizumatsu et al., 2003; Rola et al., 2004; Limoli et al., 2007; Parihar et al., 2014a). These changes occur along with reductions in dendritic complexity and synaptic density of more mature neurons (Parihar and Limoli, 2013; Parihar et al., 2014b). Consequently, cranial radiotherapy causes substantial decrements in short- and long-term learning and memory function that persist well after exposure (Greene-Schloesser and Robbins, 2012; Greene-Schloesser et al., 2013). We have previously shown in rodent models that exposure to radiation leads to long lasting reductions in neural stem cell (NSC) proliferation, prolonged oxidative stress, inhibition of neurogenesis, elevated CNS inflammation and cognitive dysfunction (Acharya et al., 2009, 2010, 2011, 2014a,c; Lan et al., 2012; Parihar et al., 2014a). While these factors are likely to contribute to the disruption of CNS function, our current understanding of the molecular and cellular mechanisms underlying radiation-induced damage in the brain, and how they impact neurocognition, are limited.

Astroglial activation is known to be a major consequence of radiation-induced chronic injury (Zhou et al., 2011; Ballesteros-Zebadua et al., 2012; Osman et al., 2014). Astrocytes form complex networks by contacting thousands of synapses and any disruption of astrocytic function following exposure to radiation will disrupt the global homeostasis of the brain (Giaume et al., 2010; Pannasch et al., 2011). Astrocytes also constitute a ‘sink’ for the metabolic clearance of neurotransmitters and the signaling molecule adenosine (Boison, 2007, 2008, 2009, 2013; Halassa et al., 2007). Adenosine is a ubiquitous modulator of synaptic transmission and neuronal activity, exerting its functions via activation of G_i/o_ protein coupled- A_1_ and A_3_ and G_s_ coupled A_2A_ and A_2B_ receptors (Boison, 2009, 2013; Boison et al., 2010; Diogenes et al., 2014). Mechanisms and physiologic functions of adenosine receptors have extensively been studied (for details see: Boison, 2013). A shift in the A_1_/A_2A_ receptor ratio/activation during radiation-induced CNS injury may reinforce the excitatory tone at synapses and contribute to cognitive dysfunction. Therefore, adenosine regulates global brain function under normal physiological settings and under pathophysiological conditions to provide neuroprotection.

Due to the widespread distribution of adenosine receptors in the brain, a tight regulation of endogenous levels of adenosine is a necessity (Boison, 2007, 2008, 2009, 2013). Astrocytes play a key role in regulating the levels of extracellular adenosine through cytosolic adenosine kinase (ADK) (Boison, 2007, 2008, 2009, 2013). ADK, which phosphorylates adenosine to 5′-AMP, is considered to be the key metabolic enzyme for the regulation of extracellular adenosine in the brain (Lloyd and Fredholm, 1995). Thus, inhibition or knockout of ADK leads to rapid increases in extracellular adenosine, while overexpression of ADK leads to a reduction of the synaptic adenosine tone. During astrogliosis, ADK is overexpressed, thereby limiting the availability of synaptic adenosine that fosters neurodegeneration (Boison, 2007, 2008, 2009, 2013). Thus, metabolic regulation of adenosine by astroglial-synaptic compartments directly impacts neuronal plasticity. Importantly, relatively little is known about the impact of radiation-exposure on adenosine metabolism, astroglial and synaptic function and its correlation with cognitive function. We hypothesized that adenosine-dependent metabolic regulation is a key mechanism in ionizing radiation-induced neurodegeneration and cognitive dysfunction. Using a specific ADK inhibitor, this *proof-of-principle* study delineates the protective role of adenosine to attenuate radiation-induced cognitive decline.

## Materials and Methods

### Animals, Irradiation and 5-ITU Treatment

All animal procedures described are in accordance with NIH guidelines and approved by the University of California Institutional Animal Care and Use Committee. Four month old male athymic nude (ATN) rats (Cr:NIH-Foxn1rnu, strain 316; Charles River, San Diego) were maintained in sterile housing conditions (20°C ± 1°C; 70% ± 10% humidity; 12 h:12 h light and dark cycle) and had free access to sterilized diet and water. Rats were divided into 4 experimental groups (8–10 animals per group): 0 Gy receiving vehicle (Con), 0 Gy receiving 5-iodotubercidin, 5-ITU (Con+5-ITU), 10 Gy head-only irradiation receiving vehicle (IRR) and 10 Gy head only irradiation receiving 5-ITU (IRR + 5-ITU). Animals showing signs of eye infection and/or neophobic behavior were excluded from the study. In order to augment adenosine signaling in the brain we decided to use a pharmacological approach using the well-characterized ADK inhibitor 5-iodotubercidin (5-ITU), which induces mild and transient sedation after brain penetration. The use of a pharmacological agent allows us to test a possible clinical route of therapeutic adenosine augmentation and to prepare for future cell transplantation approaches, which will require the use of immunocompromised ATN rats, used in the present study. The ADK inhibitor 5-ITU (HY-15424, NSC 113939, MedChem Express, Princeton, NJ, USA) was made up fresh daily by dissolving in saline with 2% ethanol (v/v, Sigma, St. Louis, MO, USA). Animals received either vehicle (2% ethanol in saline, i.p.) or 5-ITU daily (3.1 mg/kg, i.p.) for 6 days in order to precondition the brain with neuroprotective adenosine. One hour after the last 5-ITU injection, animals received 0 or 10 Gy head-only X-rays. For cranial irradiation, animals were anesthetized with isoflurane (5% for induction and 2% for maintenance of anesthesia), placed ventrally on the treatment table (XRAD 320 irradiator, Precision X-ray, North Branford, CT, USA) without restraint, and positioned under a collimated (1.0 cm^2^ diameter) beam for head-only irradiation delivered at a dose rate of 1.10 Gy/min. 5-ITU dosing was based on our previous studies (Fedele et al., 2005; Williams-Karnesky et al., 2013). Neither irradiation nor 5-ITU treatment resulted in a change in the body weight of animals. All behavioral and immunohistochemical analyses were carried out at 1 month post-irradiation.

### Behavior Testing

To determine the effect of 5-ITU treatment on radiation-induced alteration in hippocampal- and frontal cortex-dependent cognition, rats from each group were subjected to cognitive testing 1 month after irradiation. Behavioral testing was conducted over 3 weeks and included two open arena, spontaneous exploration tasks (novel place recognition, NPR and NOR) followed by a fear conditioning (FC) task. Behavioral testing closely followed our previously described protocols (Acharya et al., 2009, 2015a; Christie et al., 2012) in immunocompromised animals. To avoid infections in our strain of rats, we avoided water-based test paradigms such as the Morris water maze and opted for NOR and NPR as open arena tests. All behavioral testing data were collected by independent, blinded observers and the average of these data was used to compute the results for each task. Animals were first subjected to the NPR task followed by the NOR task. For the NOR and NPR tasks, the ‘head direction to zone’ function in Ethovision XT (Noldus) was used to track object exploration. An animal was considered to be exploring an object when its head was oriented toward it and its nose was within a 1-cm radius. All experimenters were blinded to the experimental condition and animal identification. Furthermore, an additional observer blinded to all experimental conditions re-scored the behavioral data (video files) thereby confirming the automated tracking results of Ethovision XT independently. The average of both scores was used to compute all behavioral data. We did not observe animals climbing on the object or any neophobic behavior. NPR and NOR data are presented as a discrimination index (DI) and calculated as ([Novel location exploration time/Total exploration time] – [Familiar location exploration time/Total exploration time]) × 100. A positive index indicates that rats spent more time exploring novelty (i.e., switched objects or locations), while a negative score indicates that rats exhibited little or no preference for exploring novelty. The FC task was administered in three sequential phases over 3 days including a training phase, a context test and a cue test as described previously (Christie et al., 2012; Acharya et al., 2015a).

### Immunohistochemistry

Following completion of behavioral testing, animals were euthanized and perfused (intracardiac) with 4% paraformaldehyde (Acros Organics) made in phosphate buffered saline (100 mM, pH 7.4, Gibco), brains were cryoprotected (10–30% sucrose gradient) and sectioned coronally (30 μm thick) using a cryostat (Leica Microsystems, Germany). For the dual-immunofluorescence analysis of ADK and glial fibrillary acidic protein (GFAP), the following antibodies were used: rabbit anti-ADK (from the same batch that was previously characterized and validated on knockout tissue; Gouder et al., 2004), mouse anti-GFAP (EMD Millipore), goat anti-rabbit or anti-mouse conjugated with Alexa Fluor 488 or 594 (Life Technologies/Invitrogen) and DAPI (Sigma–Aldrich). Representative sections (3–4 sections/animal, four animals/group) through the middle of the hippocampus were selected for staining and stored in Tris-buffered saline (TBS, 100 mM, pH 7.4, Sigma–Aldrich) overnight. Free floating sections were first rinsed in TBS followed by Tris-A (TBS with 0.1% Triton-X-100, Sigma–Aldrich), blocked with 10% normal goat serum (NGS with Tris-A, Sigma–Aldrich) and incubated overnight in a mixture of rabbit anti-ADK (1:3000) and mouse anti-GFAP (1:500) antibodies prepared in 3% NGS and Tris-A. The next day, the sections were treated with a mixture of goat anti-rabbit Alexa Fluor 488 (1:750) and goat anti-mouse Alexa Fluor 594 (1:500 dilution each) made with Tris-A and 3% NGS for 1 h. The sections were light protected, washed with Tris-A, and counterstained with DAPI nuclear dye (1 μmol/L in TBS, 15 min) for visualization of hippocampal morphology. Immunostained sections were rinsed in TBS and mounted on clean gelatin coated slides using SlowFade Anti-fade Gold mounting medium (Life Technologies/Invitrogen). ADK positive cells were visualized under fluorescence as green and GFAP as red fluorescence.

#### Confocal Microscopy, Image Processing and 3D Quantification of Immunoreactivity

Immunostained sections were imaged using a laser-scanning confocal microscope (Nikon Eclipse Ti C2) equipped with a 40× PlanApo oil-immersion lens (1.3 NA) and an NIS-Elements AR module (v4.30, Nikon). 30 z stacks (1024 bit depth) at 1 μm from three different fields (318 μm × 318 μm area) in each section were imaged from the dentate gyrus. ADK immunofluorescence was imaged with 493 nm excitation and 518 nm emissions and GFAP was imaged with 592 nm excitation and 617 nm emissions. Images were deconvoluted using the AutoQuant software (version X3.0.4, Media Cybernetics, Rockville, MD, USA) with 1.26867 × 1.26867 × 1 μm spacing, and wavelengths set at 447 nm (DAPI), 510 (ADK) and 594 nm (GFAP). An adaptive, 3D blinded deconvolution method was used (**Figure 3**). AutoQuant automatically creates and stores deconvoluted images for direct import into the Imaris module (version 8.1.2, Bitplane, Inc., Zurich, Switzerland). The 3D algorithm-based surface rendering and quantification of fluorescence intensity for ADK and GFAP were carried out in Imaris at 100% rendering quality. Each channel was analyzed separately. 3D surface rendering detects immunostained puncta (ADK) or cell processes [GFAP, satisfying pre-defined criteria, verified visually for accuracy] (**Figure 3**). A channel mean intensity filter was applied and minimum thresholds were used for all the experimental groups. The pre-set parameters were kept constant throughout the subsequent analysis of ADK and GFAP immunoreactivity. The quantification of astrocyte number (GFAP co-labeled with DAPI) was facilitated using the Co-localization and the Spot tools of Imaris module. ADK and GFAP data was expressed as a mean immunoreactivity (percentage) relative to unirradiated controls. The method is summarized in **Figure 3**.

### Statistical Analysis

Statistical analyses were carried out using GraphPad Prism (v6). One-way ANOVA was used to assess the normal distribution of data and significance between control and irradiated groups receiving either vehicle or 5-ITU treatment. When overall group effects were found to be statistically significant, a Bonferroni multiple comparisons test was used to compare the IRR with individual experimental groups. For analysis of FC data, repeated measures two-way ANOVA were performed. All analyses considered a value of *P* ≤ 0.05 to be statistically significant.

## Results

### ADK Inhibition and Cognitive Function

#### Novel Place Recognition (NPR)

**FIGURE 1:**
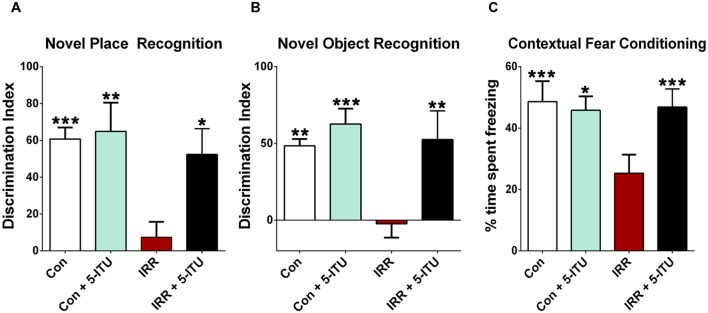
**Adenosine kinase (ADK) inhibition by systemic 5-iodotubercidin (5-ITU) treatment protects against radiation-induced cognitive dysfunction.** Adult rats received 5-ITU (3.1 mg/kg, i.p., daily for 6 days) and were irradiated (0 or 10 Gy, head only) 1 h after the last injection. Animals were divided into four experimental groups: 0 or 10 Gy whole brain irradiated receiving either vehicle or 5-ITU (Con, Con + 5-ITU, IRR, IRR+5-ITU). **(A,B)** 1 month post-irradiation, animals were tested on spatial and episodic memory retention using the NPR and NOR tasks followed by fear conditioning (FC). The tendency to explore a novel place (NPR) or object (NOR) was derived from the Discrimination Index (DI). **(A,B)** Whole brain irradiation (IRR) shows significant behavioral deficits on NPR and NOR tasks compared to controls (Con and Con + 5-ITU) as indicated by impaired preference to a novel place or object. Irradiated animals treated with 5-ITU (IRR + 5-ITU) show significant preference for the novelty when compared with irradiated (IRR) animals receiving vehicle. **(C)** 5-ITU treatment also improves behavior on the hippocampal-dependent contextual FC task. The baseline freezing levels were comparable across groups, and all groups showed elevated freezing behavior following a series of 5 tone-shock pairings. The context test was administered 24 h later, and IRR animals showed significantly decreased freezing compared to controls (Con and Con + 5-ITU). Irradiated animals receiving 5-ITU showed a significant elevation in freezing behavior that was indistinguishable from the Con group. Data are presented as mean ± SEM. (*N* = 8–10 animals/group). *P*-values are derived from ANOVA and Bonferroni’s multiple comparisons test. ^∗∗∗^*P* < 0.001; ^∗∗^*P* < 0.01; ^∗^*P* < 0.05 compared with the IRR group.

One month post-IRR, rats were habituated in an open field arena and then tested on the NPR task (**Figure 1A**). The ability to explore a novel spatial location on the NPR task is dependent on intact hippocampal function (Save et al., 1992; Mumby et al., 2002; Barker et al., 2007; Barker and Warburton, 2011). The total exploration of both objects during the familiarization and test phases were comparable between all groups for this task. The DI was calculated to measure preference or indifference for exploring novelty. A positive DI indicates a preference, or more time exploring the novel place, while a negative DI indicates indifference, or more time exploring the familiar object. Following a 1 h retention interval between the familiarization and test phases, a significant overall group effect was found for the DI [*F*_(3,28)_ = 5.88, *P* = 0.003] that differed between the groups. In the test phase, IRR animals spent significantly less time exploring the novel place compared to Con (*P* = 0.001), Con + 5-ITU (*P* = 0.01) and IRR + 5-ITU groups (*P* = 0.05, **Figure 1A**). Unirradiated animals receiving vehicle (Con) or 5-ITU (Con + 5-ITU) treatment showed comparable novel place exploration. Furthermore, after the 1 h retention interval, irradiated animals treated with 5-ITU (IRR + 5-ITU) did not differ from either Con or Con + 5-ITU animals. These data indicate that ADK inhibition by 5-ITU treatment prior to cranial IRR improved object location exploration on the NPR task as compared to irradiated animals receiving vehicle.

#### Novel Object Recognition

After NPR testing, rats were habituated and then tested on the NOR task 1 month post-IRR (**Figure 1B**). Impairment in prefrontal cortex and hippocampal function manifests as an inability to discriminate a novel from a familiar object in the NOR task (Barker et al., 2007; Barker and Warburton, 2011). The total exploration times for both objects were not different between all experimental groups for this task. In the test phase, a significant overall group difference was found between the four cohorts for the DI [*F*_(3,28)_ = 8.95, *P* = 0.001]. After a 5 min retention interval between the familiarization and test phases, Con and Con + 5-ITU rats showed a preference for the novel object (**Figure 1B**). However, irradiated rats receiving vehicle (IRR) showed a significantly diminished preference to explore novel object compared to either Con or Con + 5-ITU animals (*P* < 0.01). The novel object exploration for the Con and Con + 5-ITU animals did not differ. 5-ITU treated irradiated animals (IRR + 5-ITU) exhibited significantly improved performance on the NOR task compared to the IRR group (*P* = 0.01). The DIs for Con, Con + 5-ITU and IRR + 5-ITU groups were statistically indistinguishable. Thus, 5-ITU treatment improved novel object exploration behavior in irradiated animals.

In summary, for each of the open arenas, episodic memory tasks (NPR and NOR), a preference toward novelty (as indicated by DI) was found to be significantly greater for Con, Con + 5-ITU and IRR + 5-ITU groups in comparison with IRR group (**Figures 1A,B**), demonstrating the protective effect of ADK inhibition. These spontaneous exploration tasks (NPR, NOR) rely on the innate curiosity of an animal to explore a ‘new object placement’ or a ‘new object.’ Other factors such as fatigue, depression and/or anxiety may also affect the overall performance on these tasks, although differences in exploration during either the habituation or familiarization phases of these tasks were not found. To account for these possible confounds, animals were subsequently tested in the FC task to interrogate hippocampal function using a task not reliant on spontaneous exploration.

#### Contextual Fear Conditioning (FC)

Three distinct phases of the FC task – training, context and cue – were administered over 3 days. Group means and 95% CIs for the post-training and context phases freezing (percent) were as follows: Post-training Con (mean = 97.6, 95% CI = 95.0–100.2); IRR (mean = 94.56, 95% CI = 88.66–100.5); Con + 5-ITU (mean = 80.32; 95% CI = 69.10–91.54); IRR + 5-ITU (mean = 82.17; 95% CI = 66.35–97.98); Context Con (mean = 50.71, 95% CI = 32.88–68.54); IRR (mean = 20.68, 95% CI = 9.34–32.02); Con + 5-ITU (mean = 45.81, 95% CI = 34.78–56.83); IRR + 5-ITU (mean = 51.11, 95% CI = 39.47–62.75). Repeated measure (RM) ANOVA for the context phase (**Figure 1C**) revealed significant differences between IRR and Con groups (*P* = 0.001); between IRR and Con + 5-ITU groups (*P* = 0.001) and between IRR and IRR+5-ITU groups (*P* = 0.001). Groups did not differ significantly in the freezing behavior across baseline, post-training, pre-cue and post-cue phases (data not shown), indicating a selective deficit on the hippocampal-dependent contextual memory phase of the task (Phillips and LeDoux, 1992; Winocur et al., 2006). Irradiation did not impair motor or sensory function, since all groups demonstrated significant increases in freezing behavior after the tone-shock pairings (post-training phase). Group means and 95% CIs for the cue phase (percent time freezing) were: Con (mean = 91.53, 95% CI = 82.36–100.7); IRR (mean = 85.71, 95% CI = 66.72–104.7); Con + 5-ITU (mean = 84.48; 95% CI = 73.56–95.41); IRR+5-ITU (mean = 93.86; 95% CI = 86.02-101.7). Thus, intact amygdala-dependent cued memory acquisition of the tone-shock pairing was not impaired, and that the deficit was specific to the hippocampal-dependent contextual memory (**Figure 1C**) in which the pairing was learned.

### Radiation-Induced Elevation in ADK and Astrogliosis

**FIGURE 2:**
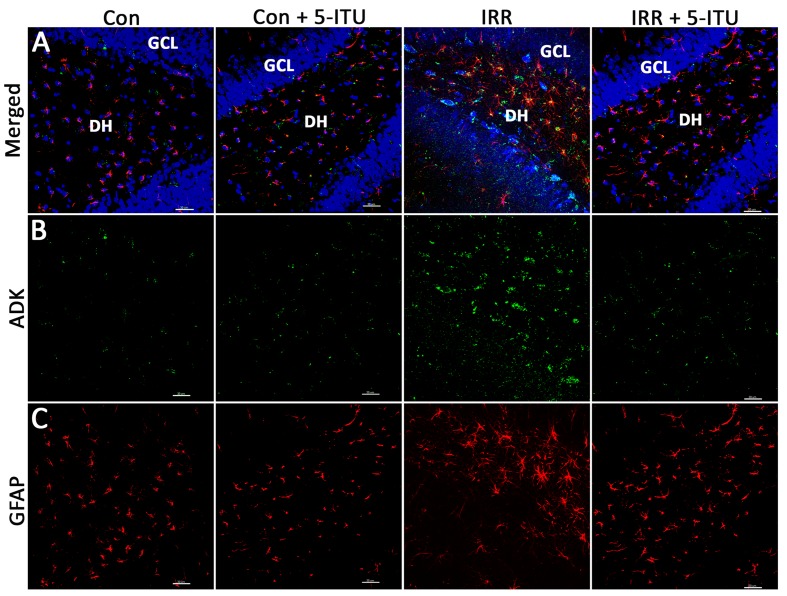
**Cranial irradiation elevates adenosine kinase (ADK) immunoreactivity and astrogliosis.** Immunofluorescence analysis demonstrates that at 1 month post-treatment, compared to controls (Con and Con + 5-ITU), exposure to cranial irradiation (10 Gy) leads to elevated ADK immunoreactivity (**A,B**; IRR group; ADK, green; DAPI nuclear counterstain, blue) that is reduced to control levels in irradiated animals treated with 5-ITU (IRR + 5-ITU). Representative confocal micrographs show the presence reactive astrocytic cell bodies (**A,C**; glial fibrillary acidic protein; GFAP, red) in the hippocampal dentate hilus (DH), sub-granular zone and granule cell layer (GCL) indicating astrogliosis. IRR + 5-ITU animals showed reduced ADK and GFAP immunoreactivity compared to IRR animals. Scale bar: 30 μm.

To assess the impact of cranial irradiation on the status of ADK and astrocytes, immunoreactivity for ADK and GFAP was assessed via dual-immunofluorescence confocal microscopy (**Figure 2**). Representative confocal micrographs revealed a marked impact of cranial irradiation on ADK and GFAP immunoreactivity. Compared to Con and Con + 5-ITU groups, irradiated animals (IRR) showed increased expression of ADK in the hippocampal granule cell layer (GCL), sub-granular zone (SGZ) and dentate hilus (DH) at 1 month post-IRR (**Figure 2B**). Concurrently, GFAP staining in unirradiated controls (Con and Con + 5-ITU) show morphological characteristics consistent with resting astrocytes (**Figure 2C**). Astrocytes in the IRR group display enlarged cell bodies with thicker and longer processes; this is consistent with hypertrophic, reactive astrocytes, or astrogliosis (**Figure 2C**).

**FIGURE 3:**
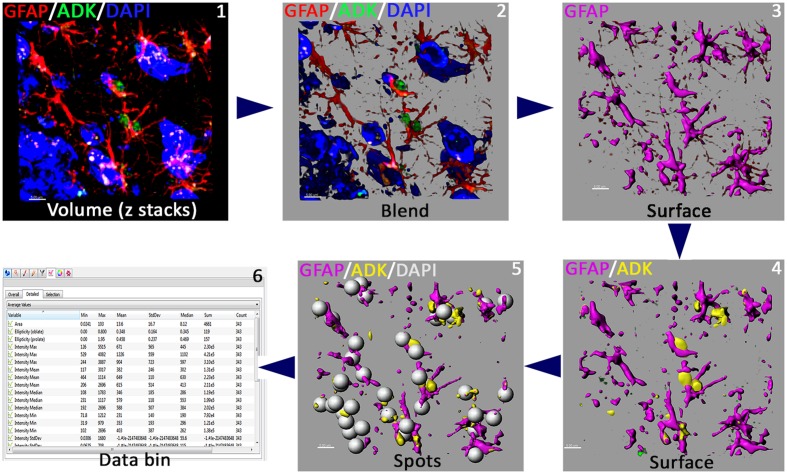
**Work flow of quantitative immunofluorescence.** Blinded deconvoluted volume of z stacks (step 1) were uploaded into Imaris (v8.1.2) for quantification of GFAP and ADK immunoreactivity (step 2). 3D algorithm-based Surface rendering of individual channels (pseudo-colored, steps 3–4) provide quantitative analyses of fluorescence intensity (GFAP, ADK) and Spot tool and Co-localization modules provide quantification of the number of astrocytes (steps 4–5). Data bin (step 6) provide quantitative immunofluorescence data for the individual channels. Scale bars: 5 μm.

**FIGURE 4:**
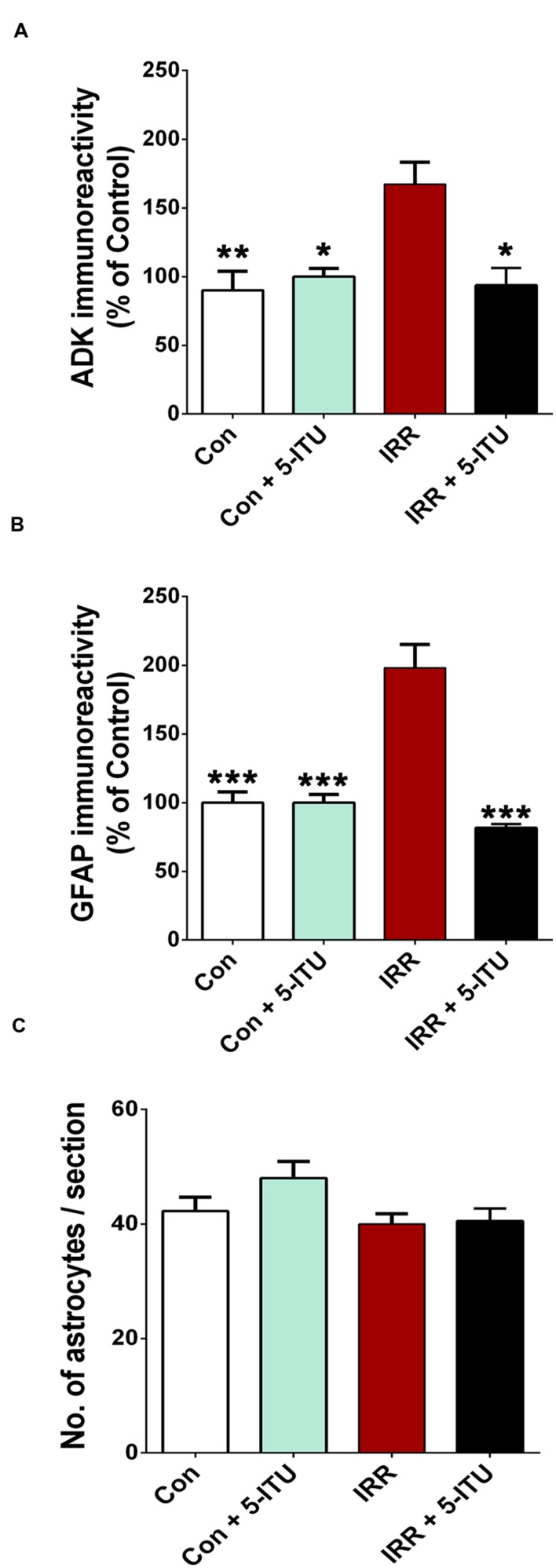
**Treatment with 5-iodotubercidin (5-ITU) attenuated radiation-induced elevation in adenosine kinase (ADK) and astrogliosis.** 3D algorithm-based deconvolution and quantification (Imaris, Bitplane, Inc.) of ADK and glial fibrillary acidic protein (GFAP) immunoreactivity show that compared to controls (Con and Con + 5-ITU), irradiation significantly increased the ADK **(A)** and astrogliotic cell bodies **(B)** in the hippocampal dentate hilus, granule cell layer, sub-granular zone and CA3/CA1 subfields. Compared with the irradiated cohort (IRR), animals receiving 5-ITU (IRR + 5-ITU) had significantly lower ADK and GFAP immunoreactivity in all hippocampal subfields. The reduced ADK and GFAP immunofluorescence was comparable to controls (Con). The number of astrocytes per hippocampal section did not change after irradiation or 5-ITU treatment **(C)**. All data are presented as mean ± SEM. (*N* = 4 animals per group). ^∗^*P* < 0.02; ^∗∗^*P* < 0.01; ^∗∗∗^*P* < 0.001 compared with the IRR group (ANOVA and Bonferroni multiple comparisons test).

High resolution, 3D algorithm-based quantification of ADK, GFAP and astrocyte number from the confocal z stacks was facilitated by blinded deconvolution (AutoQuant) and subsequent analysis using Imaris module (**Figure 3**). ADK and GFAP quantitative immunofluorescence revealed a significant increase in the immunoreactivity of the IRR group compared to controls (Con and Con + 5-ITU, **Figure 4**). Cranial radiation exposure (IRR group) significantly elevated ADK levels by 1.5 fold (*P* = 0.01 vs. Con and *P* = 0.02 vs. Con + 5-ITU group) in the hippocampus 1 month after exposure (**Figure 4A**). In parallel, hippocampal GFAP immunoreactivity was elevated by ∼2 fold in the IRR group (*P* = 0.001, **Figure 4B**) without a significant change in the total number of astrocytes (**Figure 4C**) at 1 month. However, irradiated animals receiving 5-ITU treatment (IRR + 5-ITU) showed a significant reduction in ADK immunoreactivity (*P* = 0.02) and astrogliosis (*P* = 0.001) throughout the hippocampus compared to IRR animals. These qualitative (**Figure 2**) and quantitative (**Figure 4**) data demonstrate that pharmacological ADK inhibition could substantially protect against radiation-induced neuropathology.

## Discussion

Our findings demonstrate that adenosine’s well-known protective role also extends to the attenuation of radiation-induced cognitive impairments. Our previous data demonstrated persistent, long-term cognitive impairments from 1 to 8 months following a single IRR exposure (Acharya et al., 2014b, 2015b; Parihar et al., 2014b, 2015), and suggest that global disruption of homeostatic functions in the brain combine to compromise cognitive performance over protracted post-IRR intervals. Several neurodegenerative conditions (Alzheimer’s Parkinson’s, ALS, epilepsy) share two key features with radiation-induced neuropathology: (i) astrogliosis as a histopathological hallmark and (ii) the onset of cognitive impairment (Bell and Zlokovic, 2009; Palop and Mucke, 2009; Aarsland and Kurz, 2010; Rusina et al., 2010). Astrocytes play a key role in regulating the levels of extracellular adenosine via cytosolic ADK to form a metabolic reuptake system. Our data show that cranial irradiation triggers astrogliosis and ADK overexpression 1 month after exposure that could lead to enhanced metabolic clearance of adenosine and resulting adenosine deficiency. Radiation-induced synaptotoxicity, astrogliosis, and adenosine deficiency in turn influence cognitive function (Boison and Aronica, 2015).

Our data critically test our hypothesis that adenosine augmentation prior to irradiation is protective against radiation-induced neuropathology. Animals receiving the ADK-inhibitor prior to cranial IRR were characterized by improved behavioral performance as characterized in three distinct tasks to assess cognitive function (**Figure 1**). Treatment with 5-ITU prior to irradiation (IRR + 5-ITU) prevented development of radiation-induced memory impairments on the NPR and NOR tasks at 1 month post-exposure. In contrast to irradiated rats receiving vehicle (IRR), DIs of irradiated animals with 5-ITU treatment (IRR + 5-ITU) were indistinguishable from unirradiated controls, where both controls (Con) and 5-ITU injected animals (Con + 5-ITU) showed significant preference for exploring the novel place or object. Moreover, unirradiated animals receiving 5-ITU were statistically indistinguishable from the controls receiving vehicle. In these preventative studies we chose a time point of analysis (4 weeks after irradiation), which reflects the delayed onset of cognitive dysfunction after radiation therapy (Tofilon and Fike, 2000) in combination with the prophylactic use of an ADK inhibitor. Whether, prophylactic ADK inhibition affects cognitive function at different time points post irradiation, or whether post-irradiation treatment with an ADK inhibitor might be of therapeutic benefit has not been addressed here, but might be interesting to investigate in future work.

The effectiveness of ADK inhibition to prevent radiation-induced behavioral deficits was further confirmed using the contextual FC task (**Figure 1C**) that engages the hippocampus and does not rely on spontaneous exploration (Phillips and LeDoux, 1992; Winocur et al., 2006). Irradiated animals (IRR) spent significantly less time in freezing than Con and Con + 5-ITU cohorts during the context phase of the FC task. These data suggest that irradiation disrupted long-term (24 h) memory for the tone-shock (context) association that has been shown to rely on intact hippocampal function (Phillips and LeDoux, 1992; Winocur et al., 2006). Importantly, animals treated with 5-ITU prior to irradiation (IRR + 5-ITU) exhibited intact freezing behavior, and were statistically indistinguishable from Con and Con + 5-ITU animals in their contextual fear memory. This finding indicates that radiation-induced deficits in hippocampal-dependent long-term memory function may be prevented by ADK inhibitor-induced adenosine augmentation at the time of irradiation. The amount of post-training freezing observed was comparable between all experimental cohorts, suggesting that experimental procedures did not affect initial acquisition of the conditioned freezing response and memory consolidation. Similarly, irradiation or 5-ITU treatments did not affect freezing behavior during the cue test phase, indicating intact amygdala-dependent acquisition and memory formation (Phillips and LeDoux, 1992; Winocur et al., 2006). The specific deficits observed in contextual fear memory tasks are consistent with the impairments in the NPR and NOR tasks and suggest that cranial irradiation disrupts hippocampal and frontal cortex function, and pre-IRR treatment with an ADK inhibitor prevents radiation-induced cognitive deficits.

Our data clearly show that pharmacological inhibition of ADK can prevent a decline in cognition following cranial irradiation. In the present study, we demonstrate that in the irradiated brain (IRR), ADK is co-expressed in GFAP-positive reactive astrocytes, which are characterized by a hypertrophic morphology with larger soma and increased length and width of astrocytic stellae compared to unirradiated controls (**Figures 2A,B**). The total number of GFAP^+^ astrocytes did not differ between control and irradiated groups (**Figure 4C**). These findings indicate that astrogliosis is accompanied by ADK overexpression. Quantification of ADK and GFAP immunoreactivity by high resolution confocal microscopy showed a marked rise of fluorescence intensity in the irradiated hippocampus (**Figures 4A,B**) whereas, pre-treatment with the ADK inhibitor, 5-ITU, prevented increases in ADK and GFAP immunoreactivity in the irradiated brain. It is likely that pre-treatment with 5-ITU attenuated the initial radiation-induced injury and therefore the very processes that eventually cause increases in GFAP and ADK immunoreactivity, although epigenetic mechanisms (Williams-Karnesky et al., 2013; Boison, 2016) might also be implicated. At the doses used, 5-ITU is not known to exert any cytotoxic or apoptotic effects on astrocytes (Ugarkar et al., 2000).

Chronic inflammation is a hallmark of the irradiated brain that is linked with cognitive decline (Zhao and Robbins, 2009; Moravan et al., 2011; Belarbi et al., 2013; Acharya et al., 2014c; Parihar et al., 2014a). Our results suggest that: (1) increased ADK expression is associated with astrogliosis in the irradiated brain; ADK-induced adenosine deficiency in turn may contribute to the radiation-induced neuropathology, and (2) chronic upregulation of ADK in the irradiated brain can be prevented by the transient prophylactic administration of an ADK inhibitor; this finding is in line with a lack of radiation-induced cognitive impairments. Past studies have shown that 5-ITU was effective at increasing extracellular adenosine levels in the brain (Pazzagli et al., 1995; Boison and Stewart, 2009; Boison, 2013), and supports our current findings suggesting that inhibition of ADK prior to irradiation is neuroprotective through a similar mechanism. Inhibition of ADK reduced synaptotoxicity in the hippocampus by modulating adenosine receptors, indicating an important role of ADK in the regulation of basal extracellular adenosine (Pazzagli et al., 1995; Gouder et al., 2004; Boison and Stewart, 2009; Boison, 2013). (3) Lastly, the contribution of ADK expression to radiomimetic neuropathology would favor the development of adenosine-based therapeutic interventions such as stem cell therapies to augment adenosine signaling locally via transplanted adenosine releasing cells.

## Conclusion

Our experimental data support the overall concept that a combination of neurotoxicity, astrogliosis, and elevation of ADK, resulting in a deficiency of extracellular adenosine can directly cause a broad spectrum of comorbid symptoms that are collectively present across several neurological conditions (Aronica et al., 2013; Boison and Aronica, 2015). If radiation-induced adenosine deficiency, triggered by ADK upregulation, is sufficient to precipitate neurocognitive impairments, then therapeutic adenosine augmentation (molecular, cellular or pharmacological) should ameliorate those symptoms. More work is needed to assess whether a neuroprotective treatment interferes with the therapeutic efficacy of radiotherapy or if transient treatment with ADK inhibitors post-irradiation are as effective as pre-irradiation treatments against radiation-induced CNS dysfunction. ADK inhibitors represent some of the most promising adenosine elevating agents (Kowaluk et al., 2000; McGaraughty et al., 2005; Boison, 2013). Our experimental data support the concept that such therapeutic approaches might be useful as prophylactic pre-treatment to avoid radiation-induced cognitive impairment.

## Authors Contributions

Conception and Design: JB, CL, DB, MA; Development of methodology: TL, BA, NC, AB, MA; Acquisition of data: BA, NC, AB, MA; Analysis and interpretation of data: JB, BA, CL, DB, MA; Writing, review and/or revision of manuscript: JB, TL, CL, DB, MA; Administrative, technical or material support: JB, CL, MA.

## Conflict of Interest Statement

The authors declare that the research was conducted in the absence of any commercial or financial relationships that could be construed as a potential conflict of interest.
