# Corrigendum: Adenosine Kinase Inhibition Protects against Cranial Radiation-Induced Cognitive Dysfunction

**DOI:** 10.3389/fnmol.2017.00218

**Published:** 2017-07-10

**Authors:** Munjal M. Acharya, Janet E. Baulch, Theresa A. Lusardi, Barrett D. Allen, Nicole N. Chmielewski, Al Anoud D. Baddour, Charles L. Limoli, Detlev Boison

**Affiliations:** ^1^Department of Radiation Oncology, University of CaliforniaIrvine, CA, United States; ^2^R. S. Dow Neurobiology Laboratories, Legacy Research InstitutePortland, OR, United States

**Keywords:** adenosine, adenosine kinase, astrogliosis, radiation, cancer therapy, cognition, neuroprotection

In the original article, in Figure 2 the fourth column (IRR + 5-ITU group) accidentally had incorrect photomicrographs inserted for each channel shown (Merged, ADK, and GFAP). The corrected Figure 2 appears below.

**Figure 2:**
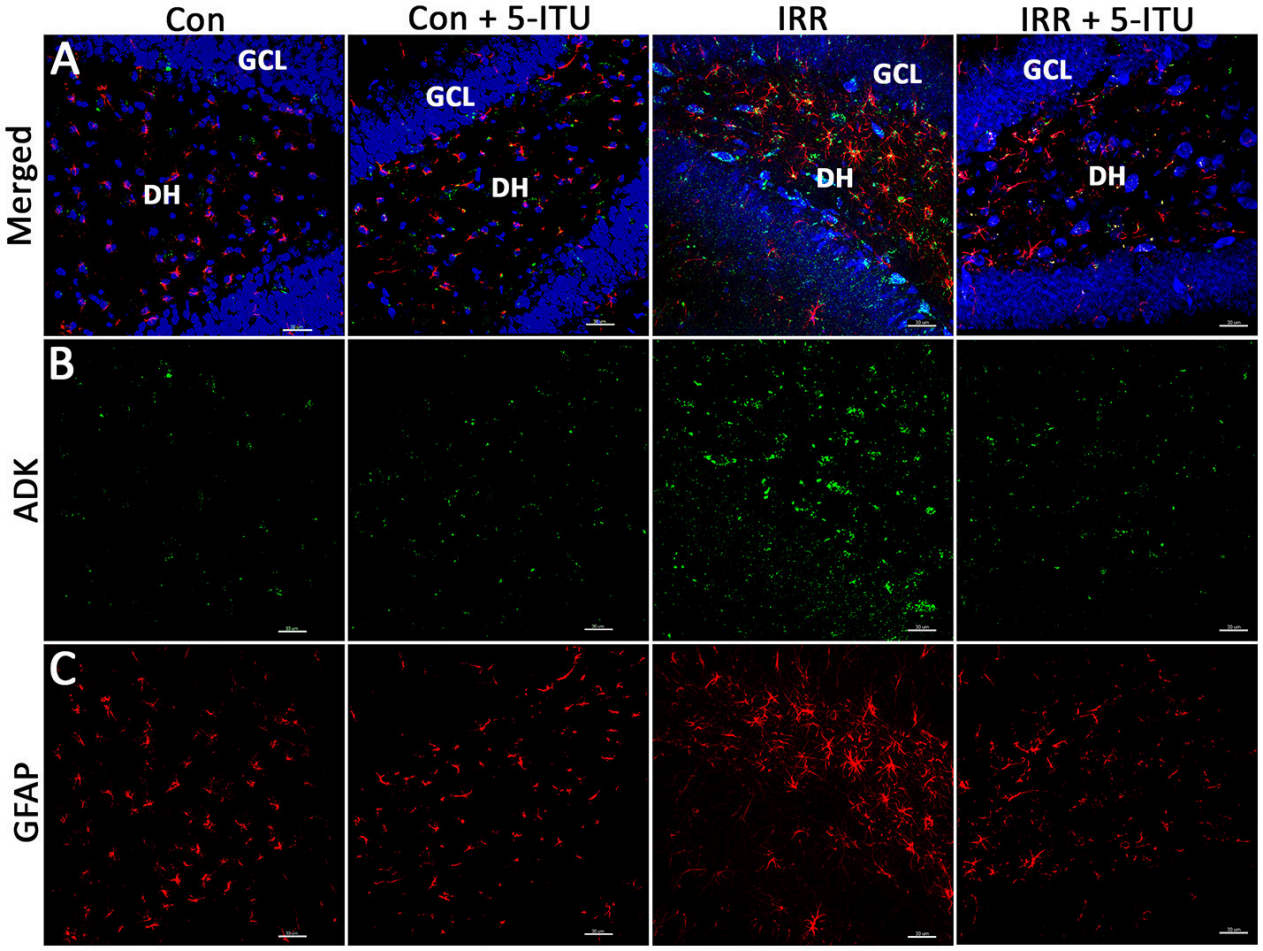
Cranial irradiation elevates adenosine kinase (ADK) immunoreactivity and astrogliosis. Immunofluorescence analysis demonstrates that at 1 month post-treatment, compared to controls (Con and Con + 5-ITU), exposure to cranial irradiation (10 Gy) leads to elevated ADK immunoreactivity (**A,B**; IRR group; ADK, green; DAPI nuclear counterstain, blue) that is reduced to control levels in irradiated animals treated with 5-ITU (IRR + 5-ITU). Representative confocal micrographs show the presence reactive astrocytic cell bodies (**A,C**; glial fibrillary acidic protein; GFAP, red) in the hippocampal dentate hilus (DH), sub-granular zone and granule cell layer (GCL) indicating astrogliosis. IRR + 5-ITU animals showed reduced ADK and GFAP immunoreactivity compared to IRR animals. Scale bar: 30 μm.

Similarly, the reference for Osman, A. M. et al. (2014; doi: 10.3727/096368913X674648) has been incorrectly cited. It should be disregarded. The authors apologize for these errors and state that this does not change the scientific conclusions of the article in any way.

## Conflict of interest statement

The authors declare that the research was conducted in the absence of any commercial or financial relationships that could be construed as a potential conflict of interest.

